# The role of SNMPs in insect olfaction

**DOI:** 10.1007/s00441-020-03336-0

**Published:** 2020-11-27

**Authors:** Sina Cassau, Jürgen Krieger

**Affiliations:** Institute of Biology/Zoology, Department of Animal Physiology, Martin Luther University Halle-Wittenberg, 06120 Halle (Saale), Germany

**Keywords:** Olfactory sensilla, Olfactory sensory neuron, Pheromone detection, Sensory neuron membrane protein, CD36

## Abstract

The sense of smell enables insects to recognize olfactory signals crucial for survival and reproduction. In insects, odorant detection highly depends on the interplay of distinct proteins expressed by specialized olfactory sensory neurons (OSNs) and associated support cells which are housed together in chemosensory units, named sensilla, mainly located on the antenna. Besides odorant-binding proteins (OBPs) and olfactory receptors, so-called sensory neuron membrane proteins (SNMPs) are indicated to play a critical role in the detection of certain odorants. SNMPs are insect-specific membrane proteins initially identified in pheromone-sensitive OSNs of Lepidoptera and are indispensable for a proper detection of pheromones. In the last decades, genome and transcriptome analyses have revealed a wide distribution of SNMP-encoding genes in holometabolous and hemimetabolous insects, with a given species expressing multiple subtypes in distinct cells of the olfactory system. Besides SNMPs having a neuronal expression in subpopulations of OSNs, certain SNMP types were found expressed in OSN-associated support cells suggesting different decisive roles of SNMPs in the peripheral olfactory system. In this review, we will report the state of knowledge of neuronal and non-neuronal members of the SNMP family and discuss their possible functions in insect olfaction.

**Supplementary Information:**

The online version contains supplementary material available at 10.1007/s00441-020-03336-0.

## Introduction

The recognition of olfactory signals is of crucial importance for survival, reproduction, and communication with conspecifics in almost all insects. As a consequence, many species of this animal group have evolved an extraordinarily powerful sense of smell enabling a highly sensitive and precise detection of informative odorants originating from odor sources as diverse as food, predators, and oviposition sites as well as conspecifics that release pheromones (Hansson and Stensmyr 2011, Andersson et al. 2015). On their body surface, insects comprise hundreds to up to several ten thousand chemosensory units, called sensilla (Steinbrecht 1996, Shanbhag et al. 1999), mainly concentrated on their antenna and found in lower densities on other body parts, such as the palps, the labellum, the legs, the wing margins, and the ovipositor. Each odor-detecting sensillum is equipped with a number of olfactory sensory neurons (OSNs) and associated support cells which jointly express the proteins that ensure a sensitive and specific peripheral recognition of relevant infochemicals.

In recent years, considerable progress has been made in enlightening the cellular and molecular basis of olfactory signal detection in insects (reviewed in Leal 2013, Kohl et al. 2015, Montagne et al. 2015, Zhang et al. 2015b, Fleischer and Krieger 2018). Support cells have been shown to express members of the odorant-binding protein (OBP) family and to secrete them into the sensillum lymph surrounding the dendritic (ciliary) extensions of OSNs. Soluble OBPs are supposed to bind odor molecules that enter the porous sensillum and mediate their protected transfer through the lymph towards ligand-matched receptors in the dendritic membrane of OSNs (Leal 2003, Pelosi et al. 2006, Brito et al. 2016). Diverse olfactory receptors are expressed across the population of OSNs of an insect and form the basis for the ability to accurately detect a variety of behavioral significant odorants, including pheromones. Each OSN generally expresses only one ligand-binding olfactory receptor type from one of the several chemosensory receptor gene families characterized in insects. Seven transmembrane domain odorant receptors (ORs) represent the vast majority of olfactory receptor types (Clyne et al. 1999, Vosshall et al. 1999), and ionotropic glutamate receptor–like ionotropic receptors (IRs) (Benton et al. 2009) are a second, larger group (Montagne et al. 2015, Wicher 2015, Fleischer et al. 2018). OR-expressing OSNs require the OR co-receptor Orco for proper function (Larsson et al. 2004, Benton et al. 2006) which heteromerizes with the ligand-binding OR forming a complex that functions as a ligand-activated cation channel required for chemoelectrical signal transduction of odorants and pheromones (Neuhaus et al. 2005, Benton et al. 2006, Sato et al. 2008, Wicher et al. 2008).

So-called sensory neuron membrane proteins (SNMPs) were discovered as abundant proteins in the dendritic membrane of pheromone-sensitive OSNs of moths (Rogers et al. 1997). Since then, they are considered to play a critical role in the pheromone detection process. The insect-specific SNMPs belong to a larger family of transmembrane receptors and transporters named according to the vertebrate protein CD36 (Rogers et al. 1997, Nichols and Vogt 2008). Several possible roles of SNMPs have been proposed based on their expression in pheromone-sensitive OSNs and their apparent evolutionary relationship to CD36 family proteins, including functions in the transmembrane transport of lipophilic compounds, in the docking of OBP/pheromone complexes to the membrane, and as co-receptors mediating the transfer of pheromones to odorant receptors (Vogt 2003).

Following the discovery of SNMPs in moths, orthologues have been identified in many insect species from various orders, utilizing available SNMP sequences in extensive homology-based searches of transcriptome and genome databases (Vogt et al. 2009, Zhang et al. 2020, Zhao et al. 2020). Within a given species, several SNMP-types have been revealed, which appeared to be differentially expressed in distinct subpopulations of the OR-expressing OSNs (Rogers et al. 2001a, Benton et al. 2007, Pregitzer et al. 2019) and (contrary to what the name SNMP implies) in the support cells of olfactory sensilla (Forstner et al. 2008, Gu et al. 2013, Blankenburg et al. 2019). While the role of SNMPs expressed by non-neuronal support cells is not yet understood, most recent results indicate that the neuronal-expressed SNMPs may act as co-receptors in the membrane of OSNs possibly involved in the transfer of lipophilic pheromones and distinct odorants to a nearby OR (Gomez-Diaz et al. 2016).

Altogether, the current data highlight the fundamental importance of SNMPs in the primary events of insect olfaction. In this review, we will give an overview on the diversity and expression of the SNMP gene family and the role of the proteins in the peripheral olfactory systems of insects. We will consider possible functions of SNMPs that are expressed in non-neuronal support cells, but direct our main focus on the relatively well-studied neuronal SNMPs. Regarding the latter, we will discuss results and current concepts on their functions in the dendritic membrane of pheromone-sensitive OSNs and their interplay with colocalized ORs and extracellular OBPs.

## The discovery and diversity of the SNMP gene family

SNMPs were discovered in Lepidoptera. Analyses of purified dendritic membrane preparations of pheromone-specific sensilla of male *Antheraea polyphemus* moths revealed an abundant 69 kDa protein which was partially sequenced. This sequence information was used to screen a cDNA library of the antenna finally leading to a full-length clone encoding the respective protein (Rogers et al. 1997). Immunohistochemical studies using antibodies raised against the bacterially expressed protein demonstrated its abundancy in the dendritic membrane of OSNs housed in male moth pheromone-sensitive sensilla (Rogers et al. 1997, Rogers et al. 2001b). Fittingly, but somewhat general, the protein was named sensory neuron membrane protein 1 (ApolSNMP1). Noteworthy, in line with the presumption of ApolSNMP1 playing a central role in pheromone detection, earlier studies by the same group in search for pheromone receptors have disclosed a 69 kDa protein (SHMP69) specific to sensory hair dendritic membrane preparations that was photolabeled by a radioactive pheromone analog (Vogt et al. 1988).

The ApolSNMP1 sequence was the starting point for the identification of orthologues in other lepidopteran species such as the moths *Bombyx mori*, *Manduca sexta*, and *Heliothis virescens* by means of homology-based cloning and screening approaches (Rogers et al. 2001a). Besides SNMP1s, the screenings revealed genes encoding proteins of 25–30% sequence identity named SNMP2s (Rogers et al. 2001a, Forstner et al. 2008). More recently, a similarly related third SNMP type (SNMP3) was reported in the genome of several moth species (Liu et al. 2015).

The rapid progress in sequencing technologies and bioinformatics tools has significantly promoted the identification of new SNMPs within and beyond Lepidoptera (Table S1). Over the last decades, BLAST analyses of genomes and transcriptomes employing available SNMPs identified candidate SNMPs in various insects of holometabolous orders, namely, Diptera (flies and mosquitoes), Coleoptera (beetles), Hymenoptera (bees and wasps), and Neoptera (lacewings), as well as hemimetabolous insects, i.e., Hemiptera (bugs), Orthoptera (locusts), and Psocodea (lice) (overview in Nichols and Vogt 2008, Jiang et al. 2016, Zhang et al. 2020, Zhao et al. 2020).

Initial naming and classification of newly identified SNMPs were mainly based on their phylogenetic relationship to the moth SNMP types, indicating a subdivision of the insect SNMP clade into three main groups (SNMP1s to SNMP3s). However, the inclusion of more and more available SNMPs into their phylogenetic analyses revealed partly new relationships between SNMPs, regrouping SNMPs originally categorized as SNMP2s with SNMP3s and vice versa (Zhang et al. 2020).

Collectively, the data document a diverse SNMP gene family with variable numbers of SNMPs in species of the same and different insect orders ranging from two SNMPs in the vinegar fly *Drosophila melanogaster* (Nichols and Vogt 2008) to 16 in the dung beetle *Onthophagus taurus* (Zhao et al. 2020). Within a species, paralogues of the same SNMP type may exist; for example, the genome of the hessian fly *Mayetiola destructor* encodes six SNMP1 paralogues, five of which are transcribed in the antennae (Andersson et al. 2014). Similarly, the red flour beetle *Tribolium castaneum* comprises paralogous genes for four SNMP1s, three SNMP2s, and two SNMP3s (Zhao et al. 2020). Paralogous genes encoding SNMP1s, SNMP2s, and SNMP3s appear to be typical for beetles as impressively demonstrated by a recent study that annotated and characterized 128 SNMPs from the genomes and transcriptomes of 22 coleopteran species. In addition, lineage-specific expansions of SNMPs were found mainly in the family Scarabaeidae defining a novel group of SNMPs (named SNMP4s) (Zhao et al. 2020).

Why certain species, such as scarab beetles or the hessian fly, have adopted so many SNMP genes during insect evolution is unclear, yet the finding suggests an expanded functional role of SNMPs in these species.

## Expression of SNMPs in the olfactory system and beyond

Analyzing the tissue-specific gene expression of the various SNMPs of an insect and characterizing the expressing cells in the antenna and other chemosensory appendages are a first step towards understanding their specific functions within in the olfactory system. In support of a central role in olfaction, the assessment of transcript levels has detected exclusive or abundant expression of the SNMP1 type in the antenna of numerous species from multiple orders, including Lepidoptera, Hymenoptera, Diptera, Coleoptera, Hemiptera, and Orthoptera (Table S1). In insects displaying paralogous SNMP1 genes, for example, the hessian fly *Mayetiola destructor* or the small brown planthopper *Laodelphax striatellus*, the transcript abundancy in the antenna differed significantly between the SNMP1 isoforms (Table S1). Beyond the antennae, pronounced levels of SNMP1 transcripts have been detected in other chemosensory appendages such as the palps or the proboscis of insects. Generally, only very low levels of SNMP1 transcripts were detected in other parts of the insect body with few exceptions in some coleopterans (high levels in the legs and abdomen) and *Drosophila melanogaster* (high levels in the legs, wings, and gut) (Table S1). Such broad expression patterns of SNMP1s might indicate that an insect uses the respective SNMP1 in an olfactory or another chemosensory context at different locations of the body.

Similar to SNMP1s, abundant transcripts of at least one SNMP2 type are found in the antenna. However, compared to SNMP1s, SNMP2s are generally more widely distributed across the insect body, as indicated by abundant transcripts in the chemosensory mouthparts, legs, wings, thorax, abdomen, and midgut (Table S1). These broader expression patterns suggest that SNMP2s, beyond being involved in olfactory processes, might serve non-olfactory functions in tissues of the insect body.

The tissue distribution of the novel coleopteran SNMP4 group (Zhao et al. 2020) still needs to be analyzed. Also, the tissue distribution of SNMP3 types has been assessed in only a few cases of moths so far (Table S1). In the beet army worm *Spodoptera exigua*, SNMP3 transcripts were found in various chemosensory and non-chemosensory tissues (Liu et al. 2015). In contrast, SNMP3 transcripts were detected only in the adult and larval gut of the silkmoth *Bombyx mori* and the cotton bollworm *Helicoverpa armigera* (Xu et al. 2020, Zhang et al. 2020); however, the specific role of the protein within the digestive system is unclear.

### Expression of SNMPs in OSNs

More conclusive support for an olfactory role of distinct SNMP types has been gained by assessing their antennal expression topography on a cellular level using in situ hybridization (ISH) and immunohistochemical (IHC) approaches.

SNMP1s have originally been discovered as proteins specific to OSNs of pheromone-sensitive sensilla in the moth *Antheraea polyphemus* (Rogers et al. 1997). Accordingly, ISH and IHC investigations of SNMP1 orthologues in other moths (Rogers et al. 2001a, Forstner et al. 2008, Gu et al. 2013, Liu et al. 2014), as well as in flies (Benton et al. 2007), wasps (Shan et al. 2020), and locusts (Pregitzer et al. 2017, Pregitzer et al. 2019), revealed their neuronal expression in OSNs of the antenna.

Colocalization studies using SNMP1s and Orco (a marker for OR-expressing OSNs) revealed that SNMP1s are co-expressed only by a subset of the OR-expressing OSNs of the antenna (Benton et al. 2007, Pregitzer et al. 2017, Pregitzer et al. 2019). Also, in locusts, SgreSNMP1 is found in a subpopulation of the Orco-expressing neurons in the palps (Lemke et al. 2020). In *Manduca sexta*, ISH studies showed that the SNMP2 is also expressed in OSNs, but in different populations than SNMP1s (Rogers et al. 2001a). A similar expression pattern was visualized for *Drosophila melanogaster* SNMP1 and SNMP2 using the respective SNMP promotors and the Gal4/UAS system to drive GFP expression in SNMP-positive OSNs (Vogt et al. 2020).

In *Drosophila melanogaster*, DmelSNMP1 is expressed in Orco-positive OSNs of trichoid sensilla which do not express IRs (Benton et al. 2009). Moreover, two-color fluorescence ISH (FISH) performed on the antenna of the hymenopteran *Microplitis mediator* using MmedSNMP1 and IR specific probes revealed non-overlapping expression patterns (Shan et al. 2020). Together, these results indicate that neuronal SNMP1s are not associated with ionotropic receptors but are confined to a subpopulation of OSNs expressing ORs and Orco.

The number and distribution of SNMP1-expressing OSNs in the antenna can vary considerably across species and between sexes. In the *Drosophila* antenna, DmelSNMP1 is expressed by each OSN of all trichoid sensilla with no apparent differences between sexes, whereas OSNs of sensilla basiconica or sensilla coeliconica are devoid of the protein (Benton et al. 2007). In *Heliothis virescens*, HvirSNMP1 is present in OSNs innervating subsets of trichoid sensilla with sex-specific differences in the number of SNMP1-expressing OSNs per sensillum (Zielonka et al. 2018, Blankenburg et al. 2019). Remarkably, in females, all the 2–3 OSNs of the trichoid sensilla express HvirSNMP1, whereas in males, only a single cell of such a cluster possesses the protein (Zielonka et al. 2018). In males, the distribution of HvirSNMP1 cells matches the expression pattern of pheromone-sensitive ORs (Gohl and Krieger 2006, Zielonka et al. 2018) and is complementary with electrophysiological recordings of male *Heliothis virescens* trichoid sensilla, demonstrating that only one OSN of the sensillum is tuned to pheromone components (Almaas and Mustaparta 1991, Baker et al. 2004). HvirSNMP1-expressing neurons in females also express pheromone-sensitive ORs (Zielonka et al. 2018), yet it is unknown whether all the SNMP1 cells of a given trichoid sensillum are tuned to pheromone components. Ultimately, these sex-specific expression patterns indicate differences in the functional impact of HvirSNMP1 between males and females of *Heliothis virescens*.

The expression of SNMP1 is not restricted to OSNs of a morphologically distinct sensillum type. In extension of the initially described ApolSNMP1 expression in OSNs of long male-specific pheromone-sensitive sensilla trichodea, immunogold labelling experiments on antennal sections of male and female *Antheraea polyphemus* localized the protein in the dendrites of OSNs innervating intermediate sensilla and basiconic sensilla (Rogers et al. 2001b). Different labelling intensities were observed for ApolSNMP1 between sensilla, indicating differences in the abundancy and functional impact of the protein in the dendrites of OSNs of various sensilla. In *Schistocerca gregaria*, SgreSNMP1 is expressed in subsets of OSNs of basiconic and likely all OSNs of trichoid sensilla (Jiang et al. 2016). Among the 20–30 OSNs of a basiconic sensillum, a considerable number were SgreSNMP1 positive, in line with the relatively high number of ORs co-expressing SgreSNMP1 in the locust (Pregitzer et al. 2019). Finally, in the wasp *Microplitis mediator*, MmedSNMP1 was immunolocalized in OSNs of sensilla placodea (Shan et al. 2020). While SNMP1 expression is documented for OSNs in various sensilla types, it is absent from olfactory sensilla coeliconica of *Drosophila* and the desert locust *Schistocerca gregaria* (Benton et al. 2007, Jiang et al. 2016). This is consistent with the finding that the OSNs of this sensillum type generally express IRs (Benton et al. 2009, Guo et al. 2013) which are not colocalized with SNMP1s.

### Expression of SNMPs in non-neuronal olfactory support cells

**Fig. 1:**
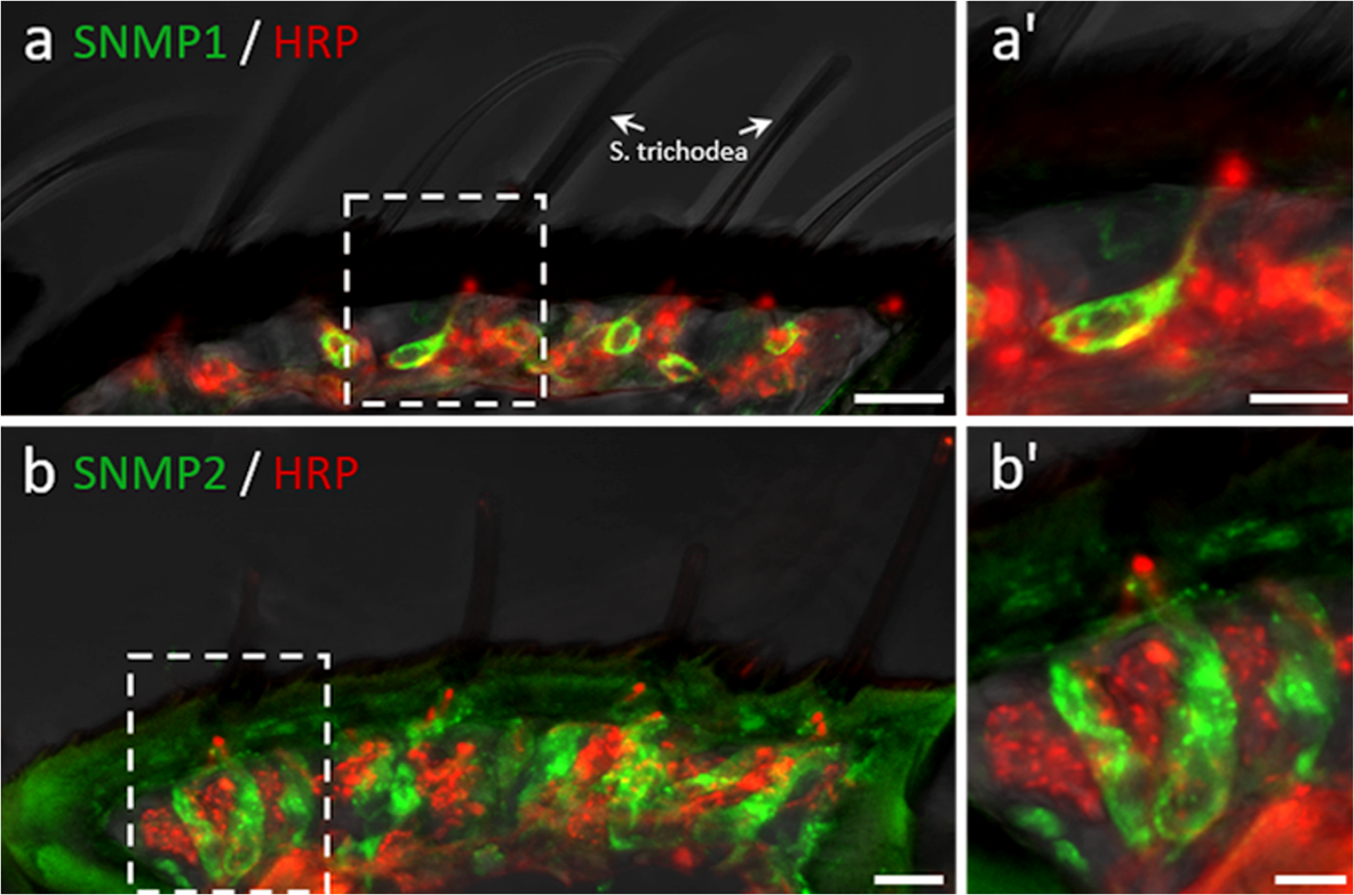
Differential expression of SNMP1 in OSNs and SNMP2 in non-neuronal support cells in the antenna of male *Heliothis virescens*. Fluorescence immunohistochemical analyses of longitudinal sections through the male antenna using antibodies specific to SNMP1 (green) and SNMP2 (green) in combination with an anti-horseradish peroxidase (HRP) antibody (red) visualizing all neurons in the antenna. Experiments were performed as described in Blankenburg et al. 2019. The upper images (**a**, a′) demonstrate the expression of SNMP1 in a subset of antennal OSNs and within distinct sensilla trichodea. Only one of the 2–3 cells of a trichoid sensillum expresses SNMP1. The lower images visualize the broad expression of SNMP2 in multiple elongated support cells of the antenna surrounding clusters of neurons (**b**, b′). The areas boxed in **a** and **b** are shown at a higher magnification on the right (a′ and b′). Scale bars: left images = 10 μm, right images = 5 μm

The SNMP1s of moths (Lepidoptera), the desert locust (Orthoptera), and the wasp *Microplitis mediator* (Hymenoptera) exhibit an OSN-specific expression. In contrast, in the antenna of these insects, the identified SNMP2 types were generally found selectively expressed in support cells surrounding OSNs (Forstner et al. 2008, Gu et al. 2013, Zhang et al. 2015a, Jiang et al. 2016, Blankenburg et al. 2019, Sun et al. 2019) revealing a differential expression of the SNMP1 and SNMP2 types and corroborating a novel function of SNMP2s in support cells. The differential expression of SNMP types became particularly obvious from studies in the moth *Heliothis virescens* (Forstner et al. 2008, Blankenburg et al. 2019), as exemplified by Fig. 1 showing an immunohistochemical analysis of sections from the antenna of a male analyzed with HvirSNMP1- and HvirSNMP2-specific antibodies. While in *Heliothis virescens*, HvirSNMP1 is expressed in a subset of antennal OSNs and within distinct sensilla trichodea OSNs (Fig. 1a, a´), HvirSNMP2 is expressed in support cells (Fig. 1b, b´), of most if not all trichoid (including pheromone-sensitive) and basiconic sensilla with no sex-specific differences (Blankenburg et al. 2019). This broad occurrence in various olfactory sensillum types, sensing a diversity of odorants as well as pheromones, indicates a more general function of the non-neuronal SNMP2 in the insect olfactory system.

The differential expression of SNMP1s and SNMP2s in OSNs and non-neuronal support cells of olfactory sensilla, respectively, suggests a functional specialization of SNMP types in insect olfaction which have been acquired and conserved in various holometabolous (moths, wasps) as well as hemimetabolous (locusts) insects during evolution. However, opposed to SNMP1s of other insects, in *Drosophila melanogaster*, expression of DmelSNMP1 has been reported in both OSNs and support cells of olfactory sensilla on the antenna (Benton et al. 2007). This finding indicates that one and the same SNMP plays a role in functionally different cell types of olfactory sensilla and may possibly serve in diverse processes. Noteworthy, reporter gene expression studies in *Drosophila* coupling the promoter of DmelSNMPs to the GAL4/UAS system to drive green fluorescent protein (GFP) expression in SNMP-positive cells revealed that DmelSNMP1 also largely associates with support cells but not neurons of taste or contact sensilla on the legs and wings whereas DmelSNMP2 was found expressed only by neurons of these sensilla (Vogt et al. 2020). These findings resemble the selective expression of SNMP types in OSNs and support cells of olfactory sensilla in other insects. In conclusion, the expression studies in *Drosophila* and other insects have drawn a complex picture with respect to the occurrence of SNMP types raising the question what different roles do SNMP1s and SNMP2s play in their respective neurons and support cells of olfactory and taste sensilla.

## Function of SNMPs expressed by OSNs

### Requirement of SNMP1s for pheromone and odorant detection

Expression studies have demonstrated the wide association of SNMP1s with pheromone-sensing OSNs (Benton et al. 2007, Forstner et al. 2008, Jin et al. 2008, Pregitzer et al. 2014). In line with a role of SNMP1 in pheromone detection, pheromone-sensitive ORs are co-expressed in OSNs with SNMP1s. For example, in the moth *Heliothis virescens*, FISH experiments have proven the colocalization of HvirSNMP1 with the pheromone receptors, HR13 and HR6, tuned to the major and minor female sex pheromone components, respectively (Krieger et al. 2002, Pregitzer et al. 2014, Zielonka et al. 2018). Similarly, in the vinegar fly, DmelSNMP1 is present in OR67d-expressing OSNs that detect the pheromone component cis-vaccenyl acetate (cVA) a compound released from the ejaculatory bulb of the males (Brieger and Butterworth 1970) and sensed by both males and females (Benton et al. 2007, Kurtovic et al. 2007).

In moths, SNMP1 expression in the antenna increases dramatically at the time point of adult emergence from pupae and continues well into later adult stages (Rogers et al. 1997, Gu et al. 2013, Liu et al. 2013, Sun et al. 2019). The onset of SNMP1 expression coincides with the time of expression of pheromone-detecting ORs in adult males’ OSNs (Gohl and Krieger 2006) and the onset of olfactory function (Schweitzer et al. 1976). Interestingly, analysis of the larval antenna of *Heliothis virescens* uncovered the presence of HvirSNMP1 in 6 out of 38 OSNs, two of which were co-expressed with the pheromone receptor HR6 (Zielonka et al. 2016). Since OSNs of the larval antenna respond to female sex pheromone components (Poivet et al. 2012, Zielonka et al. 2016), this finding indicates a critical role of SNMP1s in subsets of OSNs in earlier developmental stages and suggests a similar molecular machinery for pheromone detection in adult moths and larvae.

Yet, the function of SNMP1s appears not to be solely restricted to pheromone-sensing OSNs. In *Drosophila melanogaster*, SNMP1 is associated with OR83c, a receptor involved in the detection of the plant volatile farnesol (Ronderos et al. 2014). Furthermore, extensive FISH analyses conducted on the antenna of the desert locust *Schistocerca gregaria* revealed that out of the 83 tested ORs, 33 ORs were co-expressed with SgreSNMP1 (Pregitzer et al. 2019). While the functional aspects of these ORs have yet to be assessed and knowledge about pheromones in the desert locust is sparse, the large number of ORs co-expressed with SgreSNMP1 may suggest that not all of them are tuned to pheromones.

The essential requirement of SNMP1 for sensitive pheromone detection was first proven in 2007 using the powerful genetic model *Drosophila melanogaster* allowing for the generation of SNMP1-knockout animals. In single sensillum recordings (SSR), flies deficient for DmelSNMP1 displayed significantly diminished responses of OR67d-expressing OSNs to the pheromone cVA. These detection deficits in the SNMP1 mutants could be rescued by specific expression of DmelSNMP1 in OR67d neurons but not by the expression in support cells surrounding these OSNs, demonstrating a cell-autonomous and specific function of SNMP1 in the pheromone-sensing OSNs (Benton et al. 2007). More recently, pheromone detection deficits due to SNMP1 loss could be demonstrated for *Bombyx mori*. A deficiency of BmorSNMP1 evoked by RNA interference–based knockdown of the protein in males significantly reduced the ability of a male moth to locate the pheromone-releasing mating partner (Zhang et al. 2020).

While the requirement of SNMP1 for proper pheromone-evoked neural activity of OSNs and mating behavior have been demonstrated, the mechanisms of how SNMP1 acts in pheromone detection are largely unclear. SNMPs are glycosylated membrane proteins of about 510–560 amino acid residues (Rogers et al. 1997, Nichols and Vogt 2008, Gomez-Diaz et al. 2016). SNMPs comprise a subclade of insect genes related to a larger protein family of receptors and transporters characterized by the mammalian protein CD36 (cluster of differentiation 36) (Rogers et al. 1997, Nichols and Vogt 2008). Common for members of the CD36 gene family, SNMPs have two transmembrane domains, a large ectodomain, and rather short N- and C-terminal intracellular domains.

Insect and vertebrate members of the CD36 family exhibit diverse functions largely associated with the reception and transport of lipids and lipoproteins. For example, NINAD and Santa Maria have a role in the uptake of carotenoids in the gut and photoreceptor cells (Voolstra et al. 2006, Wang et al. 2007, Yang and O'Tousa 2007) and vertebrate CD36 family members function in lipoprotein scavenging, fatty acid transport, and lipid sensing (Silverstein and Febbraio 2009, Martin et al. 2011, Pepino et al. 2014, Oberland et al. 2015). Regarding chemosensory processes, CD36 acts as receptor for fatty acids in gustatory neurons of mammals and the zebrafish (Martin et al. 2011, Ozdener et al. 2014, Liu et al. 2017). Worthwhile emphasizing is the expression of CD36 in the cilia of a defined subset of OSNs in the nose of mice and a role indicated in the detection of oleic acid and oleic aldehyde (Lee et al. 2015, Oberland et al. 2015, Xavier et al. 2016). These findings for mouse CD36 are strikingly similar to the expression pattern of the neuronal SNMP1s in the insect antenna and their relevance for the detection of fatty acid–derived pheromones. In conclusion, they may point to analogous functions of neuronal-expressed SNMPs and CD36 in insects and vertebrates and possibly indicate an evolutionary conservation of olfactory mechanisms employed in the detection of lipophilic odorants.

### Interplay of SNMP1s with OBPs and ORs

Based on the biological activities of CD36 family members in lipid sensing and transport, potential mechanisms have been envisaged for the action of SNMP1 in pheromone detection. Already early concepts suggested SNMP1 to bind pheromone molecules alone or in the complex with OBPs thereby just concentrating pheromones in the vicinity of cognate ORs. Alternatively, a more active role as co-receptor unloading pheromones from OBPs and passing the signal molecules directly to adjacent ORs was proposed (Rogers et al. 1997, Vogt 2003). Furthermore, SNMP1 was considered to interact with intracellular proteins and to have a role in the regulation of signal transduction cascades, which is a known feature of mammal CD36 (Stuart et al. 2005, Jaqaman et al. 2011); for example, taste bud cells exhibit CD36-mediated Ca^2+^-signaling in response to low concentrations of fatty acids (Ozdener et al. 2014).

The co-receptor model implies that SNMP1 is closely associated or even organized in a complex with ORs and Orco in the ciliary membrane of OSNs, thus ensuring rapid and sensitive pheromone detection. Accordingly, yeast two-hybrid system approaches indicate protein-protein interactions of *Bombyx mori* SNMP1 with both the pheromone receptor BmorOR1 and BmorOrco (Zhang et al. 2020), as well as a protein-protein interaction between *Helicoverpa armigera* SNMP1 and the sex pheromone receptor, HarmOR13 (Xu et al. 2020). In addition, in vivo fluorescent protein fragment complementation (PCA) experiments in *Drosophila* flies (Benton et al. 2007) and Förster resonance energy transfer (FRET) assays in insect cell cultures (German et al. 2013) identified heteromeric interactions between an OR and DmelSNMP1 indicating a close vicinity of the proteins in the membrane. These findings might be a first hint of an assembly of SNMP1, ORs, and Orco in a signaling complex, thus resembling other sensory detection systems, such as insect photoreceptor cells in which a supramolecular signal complex enables efficient and fast light reception (Huber 2001, Wang et al. 2007).

Besides membrane-bound proteins, pheromone signaling involves soluble OBPs which are supposed to encapsulate pheromone molecules entering the lymph and shuttle them to the receptive elements in the dendrites of OSNs (Vogt 2003, Leal 2013). Studies in OBP-deficient moths and *Drosophila* document the essential requirement of pheromone-specific OBPs for sensitive pheromone detection (Xu et al. 2005, Ye et al. 2017, Shiota et al. 2018, Dong et al. 2019, Zhu et al. 2019). Despite substantial efforts, the mechanisms of how pheromones are released from OBPs at the sensory neuron membrane are still enigmatic (Brito et al. 2016). Binding studies with pheromones and structural analyses of OBPs at various pH values suggest a conformational change in OBPs induced by local pH changes near the membrane (Wojtasek and Leal 1999, Horst et al. 2001, Damberger et al. 2007). Whether the large extracellular domain of SNMP1s via its ionization state may contribute to a local pH environment or whether the pheromone release process might possibly involve physical interactions of OBPs with SNMP1s and/or the OR/Orco assembly, thus providing the energy for conformational changes in OBPs, awaits further investigation.

### Role of SNMP1s in OSN basal activity and odor response kinetics

While sensitive pheromone-evoked signaling clearly depends on SNMP1s and OBPs, pheromones can directly activate ORs expressed in the absence of these proteins in heterologous cell lines (Grosse-Wilde et al. 2007, Andersson et al. 2016, Yuvaraj et al. 2017) and Xenopus oocytes (Nakagawa et al. 2005, Wang et al. 2010, Jiang et al. 2014). Similarly, pheromones can induce OR-dependent responses in *Drosophila* mutants lacking SNMP1 (Li et al. 2014) or the OBP LUSH (Gomez-Diaz et al. 2013) at least when applied in high concentrations. These findings imply that pheromones must ultimately interact with ORs for OSN firing and disclose OBPs and SNMP1s as upstream elements critical for pheromone capture and delivery. Moreover, SNMP1 appears not to be required as an integral part of the molecular machinery necessary to generate the electrical response of a pheromone-sensitive neuron.

In conflict with this notion, loss of SNMP1 function due to gene defects or provoked by sensilla infusion of an SNMP1 antiserum was reported to increase the spontaneous activity of *Drosophila* OR67d-expressing neurons (Benton et al. 2007, Jin et al. 2008). Based on the elevated firing, SNMP1 was suggested as an inhibitory subunit of a receptor complex whose influence is suspended in the presence of pheromones resulting in the activation of the neurons (Jin et al. 2008, Ha and Smith 2009). However, an elevated firing of OR67d-expressing OSNs in SNMP1-deficient flies in the apparent absence of cVA was challenged by a later study suggesting that what has been interpreted as an increased “spontaneous activity” could rather represent a highly persistent ligand-induced activity initiated from exposure of grouped mutant flies to environmental cVA derived from *Drosophila* males. In support of this conclusion, SNMP1 mutant females raised in isolation from males did not display elevated spontaneous activity (Li et al. 2014). Also, considering a solely inhibitory function of SNMP1 would be rather incompatible with findings showing that the presence of SNMP1 enhances the sensitivity of responses of ORs to pheromones in heterologous cells (Benton et al. 2007, Pregitzer et al. 2014). For example, reconstitution of the OR/SNMP1 system of the moth *Heliothis virescens* in HEK293 cell lines showed that co-expression of HvirSNMP1 with the male-specific pheromone receptor HR13 increased the sensitivity of the cells to the female sex pheromone component (Z)-11-hexadecenal by about 1000-fold (Pregitzer et al. 2014).

Effective odorant sensing requires rapid responses of OSNs and then prompt inactivation after stimulus cessation. In this context, investigations of SNMP1-deficient *Drosophila* flies revealed some evidence for a decisive role of SNMP1 in controlling the signal response kinetics of pheromone-sensitive OSNs. At close-range stimulation of antennae with high cVA doses, pheromone-induced firing of OR67d neurons in SNMP1 mutant flies showed a slower activation and dramatic delay in the termination of the cVA-induced activity compared to wild-type flies (Li et al. 2014). Similarly, the OSN response kinetics differed between wild-type and SNMP1-deficient *Drosophila* when the silk moth pheromone receptor BmorOR1 was ectopically expressed in OR67d neurons and stimulated with its cognate ligand bombykol, albeit the delay in termination in the absence of SNMP1 was less pronounced. Thus, SNMP1, in addition to being required for sensitive neuronal responses to pheromones, appears to be important to achieve rapid “on/off” kinetics of receptors in response to ligands so that the receptor activation and inactivation are accelerated. Consistently, the bombykol-induced activation and deactivation of the BmorOR1/Orco receptor complex when expressed in Xenopus oocytes were accelerated when BmorSNMP1 was present (Li et al. 2014). How SNMP1 might facilitate the two opposing processes is unclear. In a model conception, SNMP1 has been suggested to act like an enzyme which can increase both the forward and reverse reaction rates by lowering the activation energy of a reversible reaction (Li et al. 2014).

### Structure function dissection of SNMP1

Insights into functionally decisive molecular features of SNMP1 have been obtained by testing *Drosophila* flies expressing SNMP1 variants bearing distinct amino acid substitutions or short deletions in the protein sequence. Through analyzing the impact of the SNMP1 modifications on the cVA response of OR67d-expressing neurons and parallel testing of the ciliary localization of the proteins, it was found that a deletion of the cytosolic N- or C-terminal tails or their substitution by corresponding domains of the CD36 family member protein (NINAD) affected neither the ciliary localization nor the functionality of SNMP1 (Gomez-Diaz et al. 2016). Thus, at least in pheromone-sensitive OSNs of *Drosophila*, SNMP1 does not seem to couple with intracellular proteins and regulate downstream pathways as was suggested from findings on mammalian CD36 (Stuart et al. 2005, Jaqaman et al. 2011, Ozdener et al. 2014).

A more detailed structure-function dissection of the SNMP1 ectodomain indicated cysteines predicted to form structure-stabilizing disulfide bonds as essential for its functionality. Moreover, several amino acids representing possible N-glycosylation sites in the extracellular domain were found critical for SNMP1 ciliary targeting and thus proper pheromone responses. Similarly, all of the 17 short deletions introduced along the length of SNMP1 ectodomain (independent from their position) led to a loss of the pheromone response, which in most cases could be ascribed to a defect in ciliary targeting of the mutated proteins. Together, the analyses of the deletion mutants underlined the functional importance of the SNMP1 ectodomain in its entirety (Gomez-Diaz et al. 2016).

First insights into how the ectodomain might act in pheromone sensing were gained from the structure homology modeling of the DmelSNMP1 ectodomain using the available 3D structure of the mammalian CD36-related protein LIMP-2 as a template (Gomez-Diaz et al. 2016). According to the 3D modeling, the SNMP1 ectodomain forms a tunnel structure large enough to accommodate pheromone molecules and to form a putative passageway that might conduct pheromones in the direction of a cognate pheromone-detecting OR in the dendritic membrane.

Due to problems with expression of the DmelSNMP1 ectodomain in bacterial or insect cell systems, binding of pheromones to the SNMP1 ectodomain could not be demonstrated yet. However, measuring the interaction of ligands with the ectodomain of the SNMP1-related mammalian CD36 protein immobilized on a self-assembled monolayer by surface plasmon resonance (SPR) could demonstrate the ability of the CD36 ectodomain to bind farnesol and insect pheromones (cVA, (Z)-11-hexadecenal, bombykol) that activate SNMP1-expressing OSNs (Gomez-Diaz et al. 2016). Moreover, consistent with a tunneling concept, replacement of putative bottle neck amino acids in DmelSNMP1 by larger residues predicted to block the tunnel passageway led to either a strong or slight loss in the pheromone sensitivity of the OR67d-expressing OSNs in the mutant flies (Gomez-Diaz et al. 2016).

### Model of SNMP1 function in pheromone sensing

**Fig. 2:**
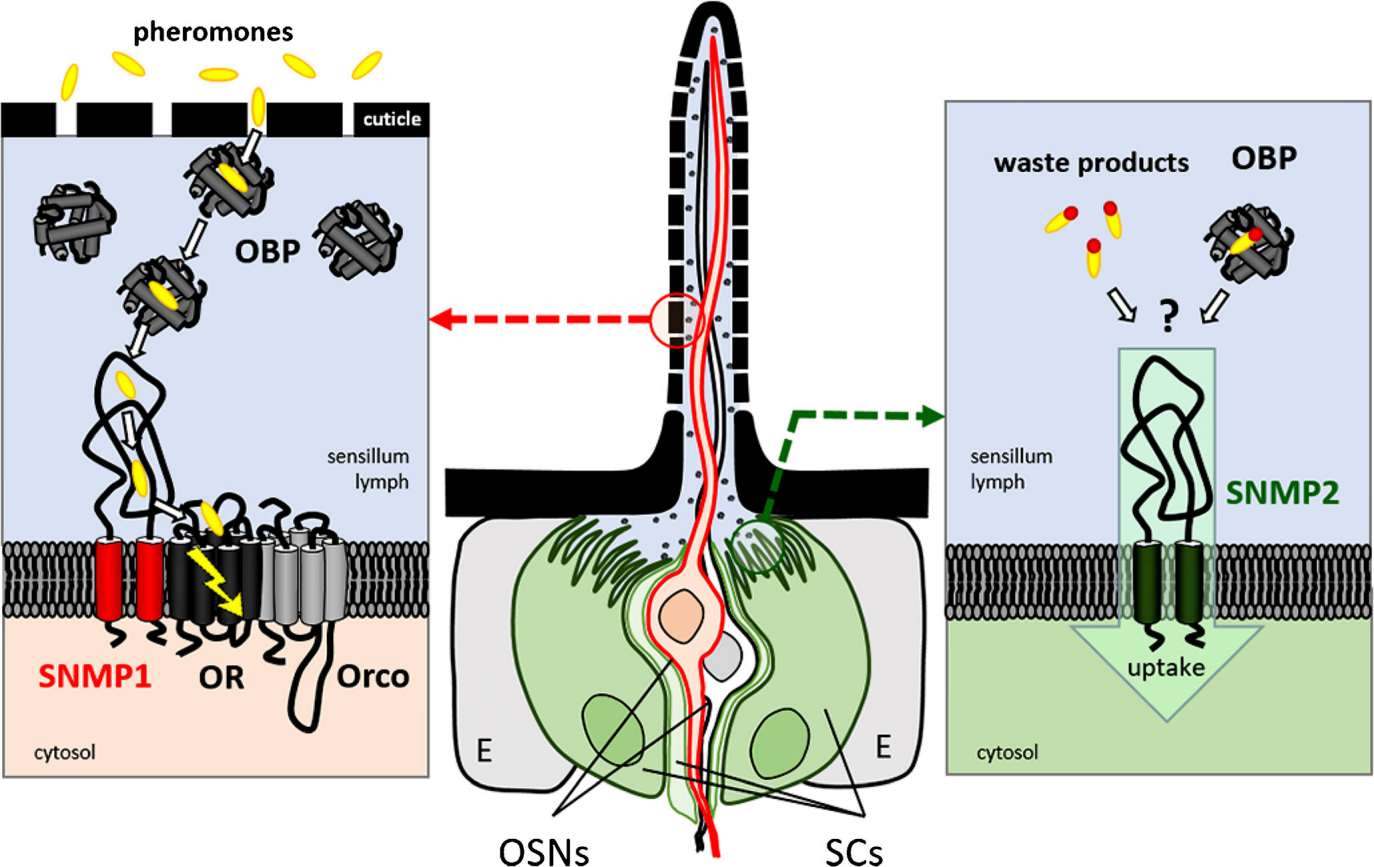
Models for neuronal SNMP1 and non-neuronal SNMP2 activities in pheromone-sensing sensilla of insects based on findings in *Drosophila* and moths. For SNMP1s, the data suggest a role in pheromone detection by distinct subsets of OSN. After pheromone molecules have entered the porous sensillum, OBPs are supposed to take up the molecules into their hydrophobic binding pocket and shuttle the encapsulated pheromone towards SNMP1 and an OR/Orco complex in the ciliary membrane of an OSNs. At the membrane, SNMP1 is supposed to mediate the release of the ligand from the OBP and to forward it via its tunnel-like ectodomain to the ligand-binding site of a pheromone-detecting OR. Ultimately, the binding of the pheromone to the OR/Orco complex triggers signal transduction. For SNMP2s, the data suggest a role in the apical microvilli membranes of support cells, contacting the sensillum lymph. Based on its localization and the transport activities of other members of the CD36 family, we proposed a function of SNMP2 in clearance processes, i.e., the uptake of “waste products” from the sensillum lymph into the support cells. In this function, SNMP2 might directly accomplish the removal of soluble breakdown products of relevant pheromones and odorants or of odorants that accidentally entered a sensillum from the lymph. Alternatively, OBPs might bind “waste products” and deliver them to SNMP2 which mediates the transfer of the OBP ligand or the ligand-OBP complex into the support cell. SCs support cells, E epidermis cells

Altogether, the SNMP1 type turns out be a critical olfactory element expressed in a subset of OSNs for sensitive and rapid detection of pheromones. In addition, a role of SNMP1 is indicated in the detection of certain non-pheromonal odorants (Ronderos et al. 2014, Pregitzer et al. 2019). The current data, mainly obtained from studying its role in pheromone sensing, propose a model in which SNMP1 acts as a co-receptor that interplays with distinct OBPs and ORs in odor detection (Fig. 2). Accordingly, pheromone molecules enter an olfactory sensillum by pores in the cuticle. At the air/sensillum lymph interface, OBPs are thought to take up and solubilize the hydrophobic pheromones in the aqueous sensillum lymph (Vogt and Riddiford 1981, Vogt 2003, Leal 2013, Brito et al. 2016). The OBP/pheromone complex is supposed to diffuse towards the receptive membrane of the OR-expressing OSN where SNMP1s in the membrane may mediate the unloading of the OBP and pave the way to the OR (Rogers et al. 1997, Vogt 2003, Gomez-Diaz et al. 2016). In this process, the SNMP1 ectodomain might trigger the release of the pheromone from the OBP, either via its ionization state and creation of a local pH that induces conformational changes in the OBP or through a direct protein-protein interaction. Subsequently, the tunnel-like ectodomain of SNMP1 could take up the pheromone and pass it on to a heteromeric OR/Orco complex that is either directly associated with SNMP1 or lies in close vicinity to SNMP1 in the membrane (Benton et al. 2007, German et al. 2013, Zhang et al. 2020). In the first case (an association of SNMP1 and OR/Orco; the case shown in Fig. 2), the pheromone could be directly transferred via the SNMP1 ectodomain tunnel to the pheromone-detecting OR ligand–binding site, supposedly lying in the transmembrane domains of the OR (Hopf et al. 2015). Considering the importance of SNMP1 for rapid activation and termination of pheromone-evoked responses (Li et al. 2014), the SNMP1 ectodomain might pipe ligands to and from the binding site of an OR. In the second case (no association of SNMP1 and OR/Orco; not shown), the tunnel might release the hydrophobic pheromones into the lipid bilayer from where they reach the OR ligand–binding site by lateral diffusion in the membrane similar to the way by which free fatty acids are suggested to approach the mammalian receptor GPR40 (Srivastava et al. 2014).

In the end, the question remains why only a subset of the OR-expressing OSNs of an insect requires SNMP1s for sensitive odorant detection, whereas SNMP1 is dispensable for robust odorant responses in the majority of the OR-expressing OSNs. Intuitively, following the current opinion that odor sensing in olfactory sensilla generally depends on OBPs and given that SNMP1 is in fact critical for ligand transfer from OBPs to ORs, one should expect that all OSNs express SNMP1. However, a recent study in *Drosophila* have challenged a general requirement of OBPs in odor detection, demonstrating robust responses of OR-expressing OSNs in six tested types of sensilla basiconica to odors of widely diverse chemical structure in the complete absence of OBPs (Xiao et al. 2019). This would suggest that in many olfactory sensilla of *Drosophila* in spite of the abundant expression of OBPs, odorants reach and activate the cognate OR-expressing neuron in an OBP-independent manner. In contrast, sensitive pheromone responses of OR67d-expressing OSNs in trichoid sensilla of *Drosophila* require the expression of the OBP LUSH (Xu et al. 2005). In the overall picture, this suggests the existence of OBP-independent and OBP-dependent odor detection processes in sensilla containing OR-expressing OSNs.

Thus, assuming that only an OBP-dependent odor detection process, as shown for pheromones (Xu et al. 2005, Ye et al. 2017, Shiota et al. 2018, Dong et al. 2019, Zhu et al. 2019), requires SNMP1 for ligand release and transfer to ORs, it is therefore possible that SNMP1 is only expressed in those subsets of OSNs which receive cognate ligands via indispensable OBPs that encapsulate the molecules to overcome the aqueous sensillum lymph. Consistent with this notion, in *Drosophila*, OSNs of sensilla basiconica that were shown to robustly respond in the absence of OBPs do not express SNMP1 (Benton et al. 2007, Xiao et al. 2019).

## Possible role of SNMPs expressed by support cells of olfactory sensilla

But what might the non-neuronal SNMP2s do? SNMP2 types exhibit a broad expression in support cells of various sensilla types of the antenna (Gu et al. 2013, Blankenburg et al. 2019, Sun et al. 2019) indicating a more general function of the protein in olfactory sensilla. Detailed immunohistochemical analyses in moths revealed a subcellular localization of SNMP2s at the apical area of support cells where microvillar protrusions border the sensillum lumen (Gu et al. 2013, Blankenburg et al. 2019). These microvilli structures are considered sites of high transmembrane transport and exchange activities (Schweitzer et al. 1976; Steinbrecht and Gnatzy 1984; Keil 1989). Therefore, the support cells are thought to secrete the sensillum lymph and to control its composition (Thurm and Küppers 1980; Steinbrecht and Gnatzy 1984). In accordance with this view, support cells secrete OBPs into the sensillum lymph and are suggested to be involved in their turnover (Galindo and Smith 2001; Klein 1987; Steinbrecht et al. 1992). Somewhat surprisingly, two independent studies in the moths *Agrotis ipsilon* and *Heliothis virescens*, which used peptide-specific antibodies directed against short regions of the extracellular domain SNMP2, found antigenicity also in the sensillum lymph–filled lumen of different sensillum types (Gu et al. 2013, Blankenburg et al. 2019). Together, these findings suggest a function of SNMP2 in the microvillar membrane of support cells and in addition might hint on an unexpected role of the proteins in the extracellular sensillum lymph.

As mentioned before, vertebrate and insect members of the CD36 gene family have activities as membrane transporters and receptors in the selective uptake of lipids, including fatty acids, carotenoids, and cholesterol, as well as of lipid-protein complexes (Koonen et al. 2005, Levy et al. 2007, Silverstein and Febbraio 2009, Raheel et al. 2019). While these activities give no plausible clue about the function of extracellularly localized SNMP2s in the sensillum lymph, they do suggest possible functions for SNMP2 in the apical microvill imembranes of the support cells. Given its location in the membrane contacting the sensillum lymph, a role of the protein as lipid or lipid-protein transporter involved in the maintenance of the extracellular fluid is conceivable (Forstner et al. 2008, Blankenburg et al. 2019). Considering the broad repertoire of ligands of mammalian CD36 (Silverstein and Febbraio 2009, Cifarelli and Abumrad 2018), SNMP2s might contribute to the elimination of various lipophilic waste products from the sensillum lymph such as breakdown products of pheromones and odorants resulting from the activity of degrading enzymes in the lymph (Vogt 2003; Leal 2013) or of odorant molecules from the air that have entered the sensillum accidentally (Fig. 2). Since certain OBPs are suggested to have a function in odor deactivation by clearing specific odorants from the sensillum lymph (Scheuermann and Smith 2019), SNMP2 might alternatively take over these OBP ligands from OBPs, a scenario resembling the proposed interaction of pheromone/OBP complexes with SNMP1. Finally, based on the confirmed role of CD36 proteins in the internalization of the fatty acid carrier albumin into dermal endothelial cells (Raheel et al. 2019), SNMP2s might mediate the uptake of waste-loaded OBPs into the support cells for further decomposition. Altogether, we suggest that similar to the multifunctional vertebrate CD36 protein, SNMP2s in support cells act as transporters and receptors for lipids as well as lipid-protein complexes. In this way, they may operate as critical membrane proteins required for the clearance of the sensillum lymph thus ensuring sensillum homeostasis and the functionality of the olfactory unit.

## Concluding remarks and perspective

Research of the last decades has emphasized the importance of SNMPs in insect olfaction. In particular, the proposed role of SNMP1s as co-receptors mediating the delivery of distinct odor molecules received from OBPs to ORs in the membrane of pheromone-sensitive OSNs has been corroborated. A different function for non-neuronal SNMP2 types, possibly in sensillum lymph clearance processes, is suggested based on their expression in support cells and a localization in the apical membrane facing the sensillum lymph. Despite this progress, a host of questions remains which await comprehensive investigations. Among the open issues are the protein-protein interactions between OBPs, SNMPs, and the OR/Orco complex and their dynamic interplay in odor detection, including proof of the proposed tunneling function of SNMP1. Also, the question to what extent does the detection of pheromonal and non-pheromonal odorants involve SNMP1s as well as the distinct roles of SNMP1 isoforms found in the olfactory system of various insect species needs examination. With respect to the proposed clearance function of SNMP2 types in olfactory sensilla, investigation towards their ligand binding and transport capabilities is urgently needed.

Experimental testing of the specific functions of the neuronal and non-neuronal SNMPs in heterologous expression systems may be technically demanding as it requires radioactively or fluorescently labeled ligands and/or OBPs as well as appropriate assay systems that permits monitoring the transient binding and dynamic transport capabilities of SNMPs. Moreover, the detailed analyses of SNMP1s need its functional reconstitution with other membrane proteins (ORs, Orco) and the involvement of soluble OBPs. While theses technical challenges have to be solved, future studies of neuronal SNMP1s and non-neuronal SNMP2s might not only illuminate their distinct roles in the peripheral olfactory processes, but might also uncover conserved and divergent mechanisms by which the paralogous proteins fulfill their specific tasks in cell types as functionally diverse as olfactory neurons and support cells of insect antenna.

## Supplementary Information

ESM 1 (PDF 1.24 mb)
