# Supplementary material for: The role of SNMPs in insect olfaction
Source: Cell Tissue Res. 2020 Nov 27;383(1):21–33. doi: 10.1007/s00441-020-03336-0 (PMC7873011; doi:10.1007/s00441-020-03336-0)
Supplement: Supplementary file 1 — (PDF 1.24 mb) [file 441_2020_3336_MOESM1_ESM.pdf]

Table S1: Expression of SNMPs in adult and larval body parts of insect species from various orders

	Insect species	SNMP type	A♂	A♀	H	Mouthparts			L	W	Th	Ab	G♂	G♀	Gut	Larva			method	source
						total	Pr	Pa								A/H	MP	Gut		
Lepidoptera	*Agrotis ipsilon*	SNMP1	★★★★	★★★	★[a]	-	★	★	★	★	☆	☆	☆	☆	-	-	-	-	qPCR	(Gu et al. 2013)
		SNMP2	★★★★	★★★	★[a]	-	★★	★★	★★	★★	☆	☆	☆	☆	-	-	-	-		
	Bombyx mori	SNMP1	★★★	★★★★	☆[a]	-	-	-	☆	☆	-	-	-	-	☆	☆	☆	☆	RT-PCR<br>qPCR	(Zhang et al. 2020)
		SNMP2	★★★	★★★★	★★[a]	-	-	-	★	★	-	-	-	-	★★	★★★	★★	★		
		SNMP3	☆	☆	★★[a]	-	-	-	☆	☆	-	-	-	-	★★★★	☆	☆	★★★★		
	Manduca sexta	SNMP1	★★★★	★★★★	☆[c]	-	-	-	☆	☆	☆	-	-	-	☆	-	-	-	NB	(Rogers et al. 2001)
		SNMP2	★★★★	★★★★	☆[c]	-	-	-	☆	☆	☆	-	-	-	☆	-	-	-		
	Antheraea polyphemus	SNMP1	★★★★	★	☆[c]	-	-	-	☆	-	-	-	-	-	-	-	-	-	NB	(Rogers et al. 1997)
	Helicoverpa armigera	SNMP1	★★★★	★★★★	☆[a]	-	☆	☆	☆	☆	-	-	-	-	☆	☆	☆	☆	RT-PCR<br>TCA<br>qPCR	(Guo et al. 2018; Xu et al. 2020b; Zhang et al. 2020)
		SNMP2	★★★★	★★★★	★★[a]	-	★★★	★★★	★★★	★★★	-	-	-	-	★	★★★	★★	★		
		SNMP3	☆	☆	☆[a]	-	-	-	☆	☆	-	-	-	-	★★★★	☆	☆	★★★★		
	Spodoptera litura	SNMP1	★★★	★★★★	-	-	☆	★	☆	-	-	-	☆	☆	-	-	-	-	RT PCR<br>qPCR	(Zhang et al. 2015)
		SNMP2	★★★	★★★★	-	-	★★	★★	★★	-	-	-	★★	★★	-	-	-	-		
	Spodoptera exigua	SNMP1	★★★	★★★★	☆[c]	-	☆	☆	☆	-	☆	-	☆	☆	-	☆	-	-	RT-PCR<br>qPCR	(Liu et al. 2014; Liu et al. 2015)
		SNMP2	★★★★	★★★★	☆[c]	-	★★	★★	★★★	-	★★★	-	★	★	-	★★★	-	-		
		SNMP3	★★	★	-	-	-	-	★	-	★	-	-	-	-	★★	-	-		
	Ectropis obliqua	SNMP1	★★★★	★★★	★[c]	-	-	-	★	★	★	★	-	★	-		★		qPCR	(Sun et al. 2019)
		SNMP2	★★★★	★★★	★[c]	-	-	-	★★	★★	★	★	-	★★	-		★			
	Sesamia inferens	SNMP1	★★★★	★★★	★[a]	-	-	-	★	★	★	★	-	☆	-		☆		qPCR<br>RT-PCR	(Zhang et al. 2013)
		SNMP2	★★★★	★★★	★[a]	-	-	-	★★	★★	★	★	-	★	-		★			
	Chilo suppressalis	SNMP2	★★★★	★★★★	-	-	-	-	★★	☆	-	-	-	☆	-	-	-	-	RT-PCR	(Xia et al. 2015)
	Mythimna separata	SNMP1	★★★★	★★★★	-	-	☆	☆	-	-	-	-	-	-	-	-	-	-	TCA	(Du et al. 2018)
		SNMP2	★★★	★★★	-	-	★★	★	-	-	-	-	-	-	-	-	-	-		
	Amyelois transitella	SNMP1	★★★★	★★★★	-	-	-	-	☆	☆	☆	☆	-	-	-	-	-	-	RT-PCR	(Leal et al. 2009)
	Streltzoviella insularis	SNMP1	★★★★	★★★★	-	-	-	-	★	-	-	-	★★	★★	-	-	-	-	qPCR	(Yang et al. 2019)
		SNMP2	★★★★	★★★★	-	-	-	-	★★★	-	-	-	★★	★★	-	-	-	-		

	Insect species	SNMP type	A♂	A♀	H	Mouthparts total	Mouthparts Pr	Mouthparts Pa	L	W	Th	Ab	G♂	G♀	Gut	Larva A/H	Larva MP	Larva Gut	method	source
Diptera	*Drosophila melanogaster*	SNMP1	★★★★	★★★★	★★★[a]	–	–	–	★★★★	★★★★	–	–	–	–	★★★★	★★★★	–	★★★★	RT-PCR	(Zhang et al. 2020)
		SNMP2	★★★★	★★★★	★★★[a]	–	–	–	★★★★	★★★★	–	–	–	–	★★★	★★★★	–	★★★		
	Hermetia illucens	SNMP1	★★★★	★★★★	–	★★	–	–	-	☆	☆	–	☆	☆	–	–	–	–	RT-PCR qPCR	(Xu et al. 2020a)
		SNMP2	★★		–	★	–	–	-	☆	☆	–	★	★★	–	–	–	–		
	Delia antiqua	SNMP1	★★★★	★★★	★[a]	-	–	–	★	★	★	★	–	–	–	–	–	–	qPCR	(Yang et al. 2020)
		SNMP2	★★★★	★★★	★[a]	-	–	–	★★	★	★	★	–	–	–	–	–	–		
	Propsilocerus akamusi	SNMP1	★★		★[a]	–	–	–	★★★★	–	–	–	–	–	–	–	–	–	qPCR	(Yan et al. 2020)
	Mayetiola destructor	SNMP1a	★	★★	–	–	–	–	–	–	–	☆	–	–	–	–	–	–	TCA	(Andersson et al. 2014)
		SNMP1b	★★★	★★	–	–	–	–	–	–	–	★	–	–	–	–	–	–		
		SNMP1c	★★	★★	–	–	–	–	–	–	–	★	–	–	–	–	–	–		
		SNMP1d	★	★	–	–	–	–	–	–	–	★	–	–	–	–	–	–		
		SNMP1e	★★★★	★★★★	–	–	–	–	–	–	–	★	–	–	–	–	–	–		
		SNMP1f	★★★	★★	–	–	–	–	–	–	–	★	–	–	–	–	–	–		
		SNMP2	★	★	–	–	–	–	–	–	–	★	–	–	–	–	–	–		
Coleoptera	*Cylas formicarius*	SNMP1	★★★		–	☆	–	–	☆	☆	–	–	–	–	☆	–	–	–	RT-PCR	(Bin et al. 2017b)
		SNMP2a	★		–	★	–	–	★	★	–	–	–	–	★	–	–	–		
		SNMP2b	★★★★		–	★★★	–	–	★★	☆	–	–	–	–	☆	–	–	–		
	Rhaphuma horsfieldi	SNMP1a	★★★	★★★★	–	–	–	–	★★	–	☆		–	–	–	–	–	–	TCA	(Zhao et al. 2020)
		SNMP1b	★	★	–	–	–	–	☆	–	☆		–	–	–	–	–	–		
		SNMP2a	★★★	★★★	–	–	–	–	★	–	★		–	–	–	–	–	–		
		SNMP2b	★	★	–	–	–	–	★★	–	★		–	–	–	–	–	–		
	Brontispa longissima	SNMP1a	★★★★	★★★★	–	–	–	–	–	–	–	★★★	–	–	–	–	–	–	TCA	(Bin et al. 2017a)
		SNMP1b	★★★	★★★	–	–	–	–	–	–	–	☆	–	–	–	–	–	–		
		SNMP2a	☆	☆	–	–	–	–	–	–	–	★★★	–	–	–	–	–	–		
		SNMP2b	★★	★★	–	–	–	–	–	–	–	★	–	–	–	–	–	–		
	Sitophilus zeamais	SNMP1a	★★★★	★★★	★[a]	–	–	–	★	★	★	★	–	–	–	–	–	–	qPCR	(Xia et al. 2019)
		SNMP1b	★★★★	★★★★	★[a]	–	–	–	★	★	★	★	–	–	–	–	–	–		
		SNMP2a	★★★★	★★★★	★[a]	–	–	–	★★★	★★★	★	★	–	–	–	–	–	–		
		SNMP2b	★★★	★★★★	★[a]	–	–	–	★★	★★	★	★	–	–	–	–	–	–		
	Anomala corpulenta	SNMP1	★★★★	★★★★	–	–	–	–	☆	–	☆		–	–	–	–	–	–	qPCR	(Li et al. 2015)

Order	Insect species	SNMP type	A♂	A♀	H	Mouthparts total	Mouthparts Pr	Mouthparts Pa	L	W	Th	Ab	G♂	G♀	Gut	Larva A/H	Larva MP	Larva Gut	method	source
Hymenoptera	*Microplitis mediator*	SNMP1	★★★★	★★★	★[a]	-	-	-	★	★	★	★	-	-	-	-	-	-	qPCR	(Shan et al. 2020)
		SNMP2	★★★	★★★★	★★[a]	-	-	-	★★★	★★	★★	★★	-	-	-	-	-	-		
	Chouioia cunea	SNMP1	★★★	★★★★	☆[a]	-	-	-	-	-	☆	☆	-	-	-	-	-	-	qPCR	(Zhao et al. 2016)
Hemiptera	*Laodelphax striatellus*	SNMP1a	★★★★	★★★★	★[a]	-	-	-	★	-	-	-	-	-	-	-	-	-	TCA	(Li et al. 2020)
		SNMP1b	★★	★★	☆[a]	-	-	-	☆	-	-	-	-	-	-	-	-	-		
		SNMP1c	★★★	★★★	★[a]	-	-	-	★	-	-	-	-	-	-	-	-	-		
		SNMP1d	★★	★★	☆[a]	-	-	-	☆	-	-	-	-	-	-	-	-	-		
		SNMP2a	★	★	★[a]	-	-	-	★★	-	-	-	-	-	-	-	-	-		
		SNMP2b	★	★	★★[a]	-	-	-	★★★	-	-	-	-	-	-	-	-	-		
		SNMP2c	★★	★★	★★[a]	-	-	-	★★★	-	-	-	-	-	-	-	-	-		
	Adelphocoris lineolatus	SNMP1a	★★★★	★★★★	-	-	-	-	-	-	★★		-	-	-	-	-	-	RT-PCR	(Xiao et al. 2017)
		SNMP1b	★★	★★	-	-	-	-	-	-	☆		-	-	-	-	-	-		
		SNMP2a	★★	★★	-	-	-	-	-	-	★★		-	-	-	-	-	-		
		SNMP2b	★	★	-	-	-	-	-	-	★		-	-	-	-	-	-		
Orthoptera	*Schistocerca gregaria*	SNMP1	★★★★	★★★	-	-	-	★★★	☆	-	-	-	-	-	-	-	-	-	RT-PCR	(Jiang et al. 2016)
		SNMP2	★★★	★★★	-	-	-	★★★	★★★	-	-	-	-	-	-	-	-	-		
	Ceracris nigricornis	SNMP1	★★★		★[a]	-	-	-	★	★	★	★	-	-	-	-	-	-	TCA	(Yuan et al. 2019)
		SNMP2a	★★★		★[a]	-	-	-	★	★	★	★	-	-	-	-	-	-		
		SNMP2b	★★★★		★[a]	-	-	-	★	★	★	★	-	-	-	-	-	-		
	Oedaleus asiaticus	SNMP1	★★★★	★★★	☆[b]	-	☆	☆	☆	☆	☆	☆	-	-	-	-	-	-	qPCR	(Zhou et al. 2019)
		SNMP2a	★★★	★★★★	☆[b]	-	☆	☆	☆	☆	☆	☆	-	-	-	-	-	-		
		SNMP2b	★	★	★[b]	-	★★★★	★★★★	★	★★★	★	★	-	-	-	-	-	-		

The data were collected from selected studies that assessed the presence and level of SNMP transcripts in tissues of body parts besides the antenna. As indicated, different methods were applied namely reverse transcriptase-PCR (RT-PCR), quantitative Real Time PCR (qPCR), Northern Blot (NB), and transcriptome analyses (TCA). The following adult body parts are represented: male antenna (A♂), female antenna (A♀), heads (H), mouthparts including a combination of all parts (total) or specifically the proboscis (Pr) and palps (Pa), legs (L), wings (W), thorax (Th), abdomen (Ab), male genitalia (G♂), female genitalia (G♀), and gut (Gut). The use of combined male and female antenna is indicated by fused "A♂" and "A♀" cells. Accordingly, data obtained for the insect body as a whole are shown by combined "Th" and "Ab" cells. The following larval body parts are represented: antenna or the head with the antenna (A/H), mouthparts (MP), and gut (Gut). The fusion of all "Larva" cells represents usage of the whole body. The stars denote the relative expression levels within a given insect and body part: ★★★★ = the highest observed transcript levels/most-intense bands. ★★★ = relatively high levels/intense bands. ★★ = low levels /moderate bands. ★ = very low levels/very faint bands. Empty stars (☆) signify that no transcript was detected. Dashes (-) indicate that the tissue was not tested. The additional superscripts within the "H" column represent the removal of any appendages: ★[a] = heads without antenna, ★[b] = heads without antenna and mouthparts, ★[c] = not specified.

Andersson MN, Videvall E, Walden KK, Harris MO, Robertson HM, Löfstedt C (2014) Sex- and tissue-specific profiles of chemosensory gene expression in a herbivorous gall-inducing fly (Diptera: Cecidomyiidae). BMC Genomics 15:501

Bin SY, Qu MQ, Li KM, Peng ZQ, Wu ZZ, Lin JT (2017a) Antennal and abdominal transcriptomes reveal chemosensory gene families in the coconut hispine beetle, *Brontispa longissima*. Sci Rep 7:2809

Bin SY, Qu MQ, Pu XH, Wu ZZ, Lin JT (2017b) Antennal transcriptome and expression analyses of olfactory genes in the sweetpotato weevil *Cylas formicarius*. Sci Rep 7:11073

Du L, Zhao X, Liang X, Gao X, Liu Y, Wang G (2018) Identification of candidate chemosensory genes in *Mythimna separata* by transcriptomic analysis. BMC Genomics 19:518

Gu SH, Yang RN, Guo MB, Wang GR, Wu KM, Guo YY, Zhou JJ, Zhang YJ (2013) Molecular identification and differential expression of sensory neuron membrane proteins in the antennae of the black cutworm moth *Agrotis ipsilon*. J Insect Physiol 59:430-443

Guo M, Chen Q, Liu Y, Wang G, Han Z (2018) Chemoreception of mouthparts: sensilla morphology and discovery of chemosensory genes in proboscis and labial palps of adult *Helicoverpa armigera* (Lepidoptera: Noctuidae). Front Physiol 9:970

Jiang X, Pregitzer P, Grosse-Wilde E, Breer H, Krieger J (2016) Identification and characterization of two "Sensory Neuron Membrane Proteins" (SNMPs) of the desert locust, *Schistocerca gregaria* (Orthoptera: Acrididae). J Insect Sci 16:33

Leal WS, Ishida Y, Pelletier J, Xu W, Rayo J, Xu X, Ames JB (2009) Olfactory proteins mediating chemical communication in the navel orangeworm moth, *Amyelois transitella*. PLoS One 4:e7235

Li X, Ju Q, Jie W, Li F, Jiang X, Hu J, Qu M (2015) Chemosensory gene families in adult antennae of *Anomala corpulenta* Motschulsky (Coleoptera: Scarabaeidae: Rutelinae). PLoS One 10:e0121504

Li Y, Hu J, Xiang Y, Zhang Y, Chen D, Liu F (2020) Identification and comparative expression profiles of chemosensory genes in major chemoreception organs of a notorious pests, *Laodelphax striatellus*. Comp Biochem Physiol Part D Genomics Proteomics 33:100646

Liu C, Zhang J, Liu Y, Wang G, Dong S (2014) Expression of SNMP1 and SNMP2 genes in antennal sensilla of *Spodoptera exigua* (Hubner). Arch Insect Biochem Physiol 85:114-126

Liu NY, Zhang T, Ye ZF, Li F, Dong SL (2015) Identification and characterization of candidate chemosensory gene families from *Spodoptera exigua* developmental transcriptomes. Int J Biol Sci 11:1036-1048

Rogers ME, Krieger J, Vogt RG (2001) Antennal SNMPs (sensory neuron membrane proteins) of Lepidoptera define a unique family of invertebrate CD36-like proteins. J Neurobiol 49:47-61

Rogers ME, Sun M, Lerner MR, Vogt RG (1997) Snmp-1, a novel membrane protein of olfactory neurons of the silk moth *Antheraea polyphemus* with homology to the CD36 family of membrane proteins. J Biol Chem 272:14792-14799

Shan S, Wang SN, Song X, Khashaveh A, Lu ZY, Dhiloo KH, Li RJ, Gao XW, Zhang YJ (2020) Molecular characterization and expression of sensory neuron membrane proteins in the parasitoid *Microplitis mediator* (Hymenoptera: Braconidae). Insect Sci 27:425-439

Sun L, Wang Q, Zhang Y, Yan Y, Guo H, Xiao Q, Zhang Y (2019) Expression patterns and colocalization of two sensory neurone membrane proteins in *Ectropis obliqua* Prout, a geometrid moth pest that uses Type-II sex pheromones. Insect Mol Biol 28:342-354

Xia D, Shen C, Zhang Y, Tang Q, Wang J (2019) Identification and expression patterns of sensory neuron membrane protein genes from *Sitophilus zeamais* Motschulsky (Coleoptera: Curculionidae). The Coleopterists Bulletin 73:735-745

Xia YH, Zhang YN, Hou XQ, Li F, Dong SL (2015) Large number of putative chemoreception and pheromone biosynthesis genes revealed by analyzing transcriptome from ovipositor-pheromone glands of *Chilo suppressalis*. Sci Rep 5:7888

Xiao Y, Sun L, Ma XY, Dong K, Liu HW, Wang Q, Guo YY, Liu ZW, Zhang YJ (2017) Identification and characterization of the distinct expression profiles of candidate chemosensory membrane proteins in the antennal transcriptome of *Adelphocoris lineolatus* (Goeze). Insect Mol Biol 26:74-91

Xu Q, Wu Z, Zeng X, An X (2020a) Identification and expression profiling of chemosensory genes in *Hermetia illucens* via a transcriptomic analysis. Front Physiol 11:720

Xu W, Zhang H, Liao Y, Papanicolaou A (2020b) Characterization of sensory neuron membrane proteins (SNMPs) in cotton bollworm *Helicoverpa armigera* (Lepidoptera: Noctuidae). Insect Sci 00:1-11

Yan C, Sun X, Cao W, Li R, Zhao C, Sun Z, Liu W, Pan L (2020) Identification and expression pattern of chemosensory genes in the transcriptome of *Propsilocerus akamusi*. PeerJ 8:e9584

Yang H, Ning S, Sun X, Chen C, Liu L, Feng J (2020) Identification and characterization of two sensory neuron membrane proteins from onion maggot (Diptera: Anthomyiidae). J Econ Entomol 113:418-426

Yang Y, Li W, Tao J, Zong S (2019) Antennal transcriptome analyses and olfactory protein identification in an important wood-boring moth pest, *Streltzoviella insularis* (Lepidoptera: Cossidae). Sci Rep 9:17951

Yuan H, Chang H, Zhao L, Yang C, Huang Y (2019) Sex- and tissue-specific transcriptome analyses and expression profiling of olfactory-related genes in *Ceracris nigricornis* Walker (Orthoptera: Acrididae). BMC Genomics 20:808

Zhang HJ, Xu W, Chen QM, Sun LN, Anderson A, Xia QY, Papanicolaou A (2020) A phylogenomics approach to characterizing sensory neuron membrane proteins (SNMPs) in Lepidoptera. Insect Biochem Mol Biol 118:103313

Zhang J, Liu Y, Walker WB, Dong SL, Wang GR (2015) Identification and localization of two sensory neuron membrane proteins from *Spodoptera litura* (Lepidoptera: Noctuidae). Insect Sci 22:399-408

Zhang YN, Jin JY, Jin R, Xia YH, Zhou JJ, Deng JY, Dong SL (2013) Differential expression patterns in chemosensory and non-chemosensory tissues of putative chemosensory genes identified by transcriptome analysis of insect pest the purple stem borer *Sesamia inferens* (Walker). PLoS One 8:e69715

Zhao Y, Wang F, Zhang X, Zhang S, Guo S, Zhu G, Liu Q, Li M (2016) Transcriptome and expression patterns of chemosensory genes in antennae of the parasitoid wasp *Chouioia cunea*. PLoS One 11:e0148159

Zhao YJ, Li GC, Zhu JY, Liu NY (2020) Genome-based analysis reveals a novel SNMP group of the Coleoptera and chemosensory receptors in *Rhaphuma horsfieldi*. Genomics 112:2713-2728

Zhou YT, Li L, Zhou XR, Tan Y, Pang BP (2019) Identification and expression profiling of candidate chemosensory membrane proteins in the band-winged grasshopper, *Oedaleus asiaticus*. Comp Biochem Physiol Part D Genomics Proteomics 30:33-44
